# Transcatheter Tricuspid Valve Intervention: Coaptation Devices

**DOI:** 10.3389/fcvm.2020.00139

**Published:** 2020-08-13

**Authors:** Guillem Muntané-Carol, Alberto Alperi, Laurent Faroux, Elisabeth Bédard, François Philippon, Josep Rodés-Cabau

**Affiliations:** Quebec Heart and Lung Institute, Laval University, Quebec City, QC, Canada

**Keywords:** tricuspid regurgitation (TR), transcatheter tricuspid intervention, FORMA, PASCAL, MitraClip device

## Abstract

Transcatheter tricuspid valve intervention (TTVI) has recently emerged as an alternative for the treatment of severe tricuspid regurgitation (TR). Multiple percutaneous devices have been developed in the last decade with promising early results. Among them, the coaptation devices are designed to reduce TR severity by valve leaflet plication or occupying the regurgitant orifice with a spacer. To date, the MitraClip/TriClip devices (Abbott, Santa Clara, CA, USA), the PASCAL system (Edwards Lifesciencies, Irvine, CA, USA), and the FORMA device (Edwards Lifesciencies, Irvine, CA, USA) have been used as coaptation devices for treating severe TR. The present document aimed to review the clinical evidence on coaptation devices in the field of TTVI, describing its design characteristics, main procedural steps, and early and mid-term outcomes.

## Introduction

**Figure 1:**
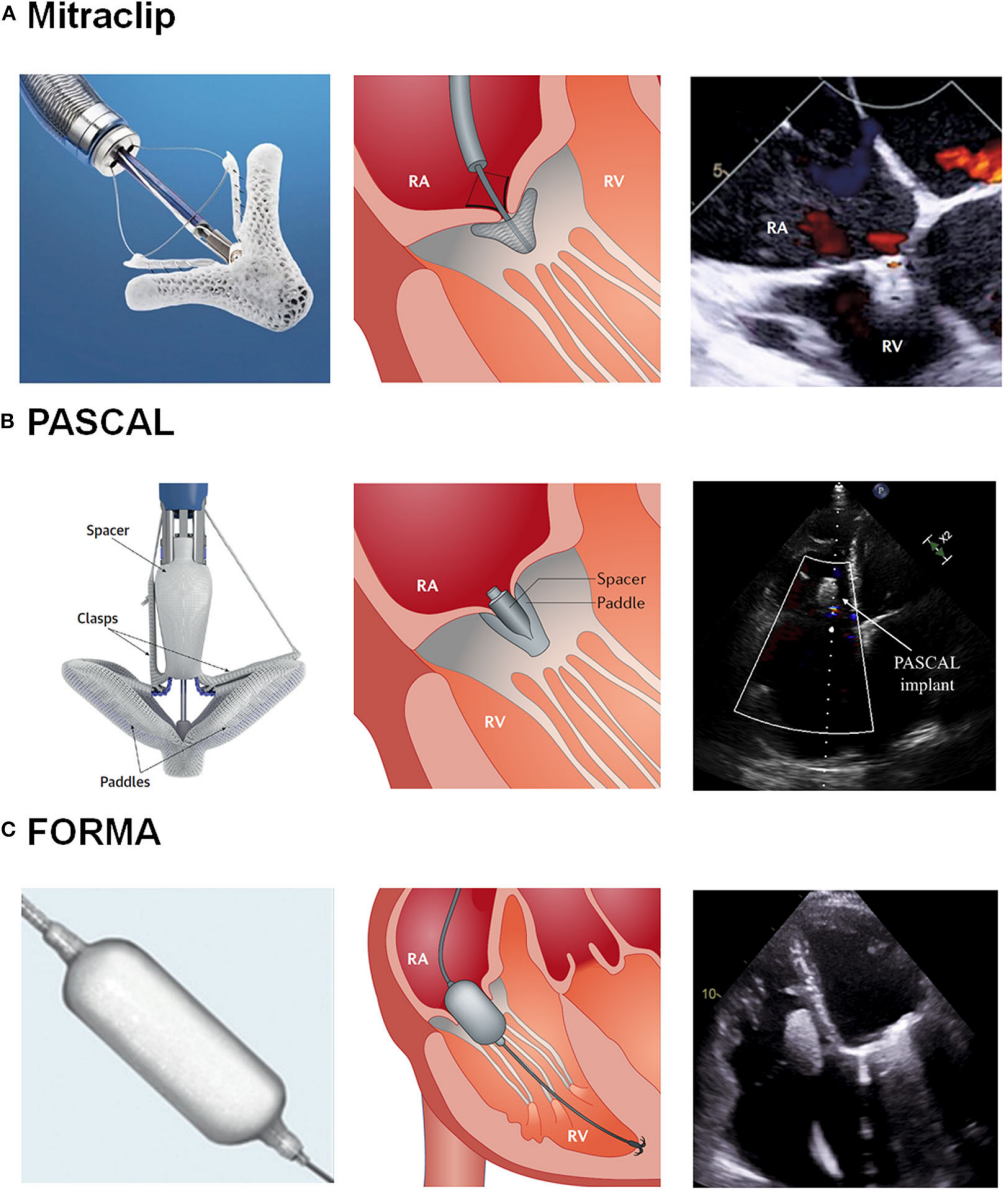
Transcatheter Tricuspid Valve Intervention: Coaptation Devices. **(A)** Illustration of the Mitraclip system and echocardiography after grasping. **(B)** Illustration of the Pascal system and echocardiography after grasping. **(C)** Illustration of the Mitraclip system and echocardiography after device implantation. RA, Right atrium; RV, Right ventricle. Reproduced with permission from Asmarats et al. (1) and Fam et al. (6) and modified for the authors.

Tricuspid regurgitation (TR) has been associated with poorer clinical outcomes as heart failure and mortality in multiple cardiology settings (1). Patients with chronic TR have been usually managed medically, leading to gradual annular dilatation and end-stage right ventricular (RV) heart failure. The etiology of TR is functional >80% of cases (1), occurring frequently in patients with already treated left-side valvular disease. Furthermore, surgical TR replacement or repair has been associated with an increased operative risk, reaching in-hospital mortality rates of about 10% (2). This higher risk is mainly related to the late referral to surgery of patients with enduring severe TR. The latter may indeed lead to an undertreated population with end-stage right heart failure, usually linked with hepatic congestion and/or late-stage kidney disease (3, 4). As a result, the management of isolated TR represents an unmet clinical need that has led to the development of transcatheter tricuspid valve intervention (TTVI) (5). TTVI can be classified according to their mechanism of action as follows: annuloplasty devices, caval stent devices, tricuspid valve (TV) replacement, and coaptation devices. The initial experience with these first-generation transcatheter systems showed that despite the high-risk profile of TR patients undergoing TTVI, the majority of the procedures were well-tolerated with low in-hospital mortality rates (lower than surgical series) (5). Among them, coaptation devices attempt to increase valve coaptation by leaflet plication or occupying the regurgitant orifice with a spacer. To date, the Mitraclip and Triclip devices (Abbott, Santa Clara, CA, USA), the PASCAL system (Edwards Lifesciencies, Irvine, CA, USA) and the FORMA device (Edwards Lifesciencies, Irvine, CA, USA) have been used as coaptation devices in the TTVI field (Figure 1) (1, 6). The present review document focuses on coaptation devices, describing its design characteristics, main procedural steps and the reported early and mid-term outcomes.

### MitraClip/TriClip System

The off-label edge-to-edge repair technique with the MitraClip system has been the most common coaptation device used in the context of TTVI, mainly due to the extensive knowledge and experience acquired in mitral regurgitation. In a recent publication from the largest worldwide retrospective series of TR patients treated with TTVI (the TriValve registry; NCT03416166), the MitraClip device was used in 80% of patients (7). Furthermore, a dedicated version of the MitraClip system to address the specific features of the TV anatomy (TriClip, Abbot, Santa Clara, CA, USA) has been recently developed (8).

#### Device Design and Procedural Planning

The MitraClip and TriClip systems consist of two parts: a steerable guide catheter (SGC) and the clip delivery system (CDS). This includes a steerable sleeve, a delivery catheter and the 4 mm (size NT) or 7 mm (size XTR) wide chrome-cobalt clip with two articulated arms to grasp and draw the valve leaflets. The 3-mm extended clip arms of the MitraClip XTR system can be useful in patients with larger coaptation gaps. The CDS are advanced through the conduit guide provided by the SGC in order to manipulate the implantable clip. In contrast to the standard MitraClip system, the SGC from the new TriClip design has two knobs for the steering maneuvers. In addition, the steerable sleeve has only one knob for tip deflection and its distal curve has a shorter radius. These iterations from the TriClip system facilitate bending and guiding maneuvers in the right atrium (RA).

**Figure 2:**
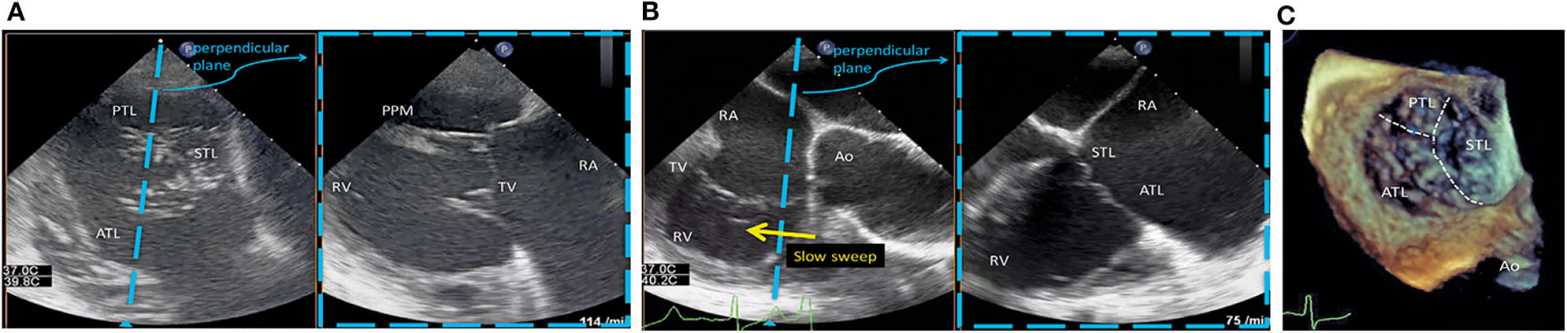
TEE protocol to patient selection for MitraClip/TriClip procedures. **(A)** Transgastric basal short-axis view. ATL, anterior tricuspid leaflet; PPM, posterior papillary muscle; PTL, posterior tricuspid leaflet; RA, right atrium; RV, right ventricle; STL, septal tricuspid leaflet; TV, tricuspid valve. **(B)** Mid-esophageal TEE view. The left view is orientated along the anteroseptal coaptation line usually at 80–90°. The right image is obtained by a sweep from the aorta to the valve center. Ao, aorta; ATL, anterior tricuspid leaflet; RA, right atrium; RV, right ventricle; STL, septal tricuspid leaflet; TV, tricuspid valve. **(C)** 3D-TEE imaging view. Ao, aorta; ATL, anterior tricuspid leaflet; PTL, posterior tricuspid leaflet; STL, septal tricuspid leaflet. Reproduced with permission from Hausleiter et al. (9).

Patient planning include transthoracic and transesophageal echocardiography (TEE). Also, a computed tomography may be performed to evaluate the venous system before the procedure. TEE is crucial (especially the transgastric basal short axis view) to establish the feasibility of the intervention. When opened to 120 degrees, the NTR and XTR clips have a width of 17 and 22 mm, respectively. The anatomical examination requires the evaluation of the leaflet length, the presence of tethering, and gaps (leaflet insertion should be located ≥6 and ≥9 mm for NTR and XTR, respectively). The leaflet gaps should be ≤7 mm. A standardized TEE protocol to help in the process of patient selection has been recently proposed (9) and can be summarized as follows (Figure 2):

1. *Transgastric basal short-axis view* (Figure 2A):
  - The only 2D view that allows simultaneous visualization of all three leaflets and commissures. Commonly obtained between 25 and 50 degrees.
  - Helpful to measure the tricuspid annulus size and assess leaflet motion and coaptation gaps.
  - The location of vena contracta width and area along with the regurgitant jet can be visualized to assess the severity of TR.
  - Useful for steering the clip intraprocedurally to the desired position within the annular plane and to rotate the clip device to achieve perpendicularity with the respective leaflet coaptation line.
2. *Mid-esophageal TEE view* (Figure 2B):
  - A slow sweep along the septal leaflet allows a detailed assessment and measurement of coaptation defects and gaps as well as leaflet length, tethering, and restriction. A restricted leaflet movement, especially of the septal leaflet, frequently limits leaflet grasping.
3. *3D-TEE imaging view (mid or lower esophageal position*, Figure 2C):
  - 3D imaging could be helpful for secondary assessment of both the clip perpendicularity to the coaptation line and the tissue bridge after grasping and clip closure.
  - An orientation *en face view* identically to the transgastric view is recommended, which places the interventricular septum on the right side of the screen with the aorta located at a five o'clock position. This facilitates peri-procedural orientation and image interpretation.

To date, a predominant central/anteroseptal jet and smaller TV coaptation gaps (cutoff of 7.2mm) have been identified as predictors of procedural success and greater TR tenting and effective regurgitant orifice area as predictors of procedural failure (10–12). Moreover, a very small and severely restricted septal leaflet is a common anatomic exclusion for the procedure (13).

The edge-to-edge repair technique in the presence of a transtricuspid cardiac implantable electronic device (CIED) may represent a more challenging subset of patients due to potential interference with the MitraClip system and acoustic shadowing. While not contraindicated, careful evaluation is critical to estimate the feasibility of leaflet grasping and to exclude the CIED *per se* as the main etiologic factor involved in the TR mechanism.

Finally, the first-in-human report using the newer third-generation MitraClip G4 system (NT, XT, NTW, and XTW) have been published very recently (14). Among other, this new system has iterations regarding the grasping area and mechanism (independent leaflet capture), along with 50% wider clip arms (NTW and XTW devices).

#### Procedural Steps

The procedure is performed under general anesthesia with fluoroscopic and TEE guidance. Whereas the first series of cases were performed via the transjugular approach (15), the first transfemoral cases were also reported early in the development of the technique (16, 17). The access through the superior vena cava provides less angulation when approaching the tricuspid annulus compared to the transfemoral approach. However, it was abandoned due to the length of the SCG, the less comfortable position for the operators, and the good performance of the system via the transfemoral approach. Moreover, the transfemoral access allows both mitral and tricuspid edge-to-edge repair in the same procedure using the same vascular approach. Following standard percutaneous femoral vein access to accommodate the SCG (24 French), unfractionated heparin is administered to achieve an activated clotting time of 250 to 350 s and the SCG is positioned in the RA.

Two methods to reduce the TR grade with the MitraClip/TriClip system have been described. First, the triple-orifice technique (TOT), where clips are ideally placed centrally between the septal and anterior tricuspid leaflet and between the septal and posterior tricuspid leaflet. Alternatively, the bicuspidization technique (BT) can be used, where clips are placed between the septal and anterior tricuspid leaflets (18). The leaflets are first approximated with an initial clip in the anteroseptal commissure and then subsequent clips are placed inward. This may allow the treatment of patients with severe RV dilatation and large coaptation defects (“zipping” technique). In accordance with this strategy, preclinical data using and *ex-vivo* model showed that the cardiac output could be increased either by placing 2 clips into the anteroseptal commissure and by the concomitant placement of clips into the anteroseptal and posteroseptal commissure (19). Finally, although the procedural results are similar between the two techniques, the BT is more feasible and has been done more frequently (12).

When using the standard MitraClip delivery system, the short distance between the inferior vena cava orifice to the atrial septum as well as the close proximity between the inferior vena cava and the tricuspid valve may difficult the steering of the MitraClip system vertical to the valve plane. To overcome this issue, the CDS is inserted into the SCG and then rotated (“mis-keyed”) by 90 degrees counter-clockwise (20). This maneuver optimizes the position of the device relative to the tricuspid annulus, allowing a clockwise rotation of the guide and enabling the “A-knob” to bend the CDS into a direction perpendicular to the tricuspid valve plane.

**Figure 3:**
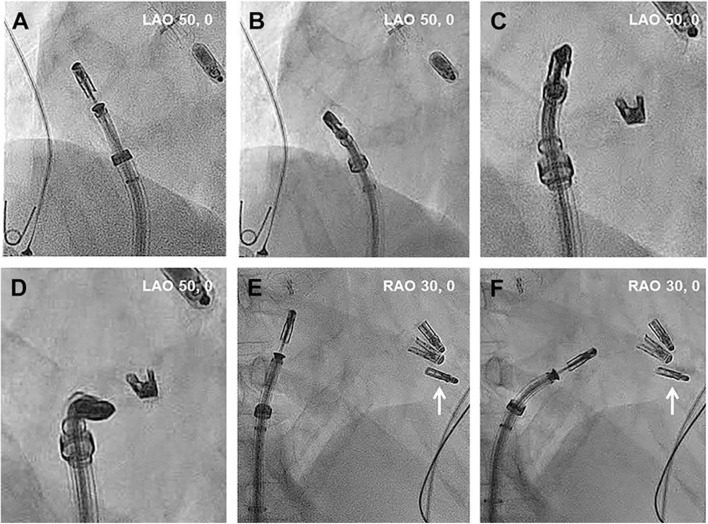
Intraprocedural main fluoroscopic views in transcatheter tricuspid valve intervention using the Mitraclip system. **(A–D)** Left anterior oblique projection. Once the CDS is straddled, turning the SGC clockwise steers away the MitraClip from the interatrial septum toward the TV. In that case, the position of the prior MitraClip placed at the TV can serve as a reference. **(E,F)** Right anterior oblique projection. Steering of the MitraClip toward the TV. The existing MitraClip is marked with white arrows. RAO, Right anterior oblique; LAO, Left anterior oblique. Reproduced with permission from Tang (21).

Once the SCG is placed in the RA, the CDS is straddled in the SGC and placed inside the RA under fluoroscopy in left anterior oblique (LAO) projection (Figure 3) (21) and echocardiographic guidance. When the clip is visualized by TEE and properly oriented (LAO fluoroscopic projection in combination with 3D TEE), the C-arm is positioned in right anterior oblique (RAO) position (fluoroscopic grasping view). Afterwards, the clip is closed to 60 degrees (to avoid entanglement with the tricuspid subvalvular apparatus) and advanced toward the right ventricle. Subsequently, the clip is opened and positioned relative to the leaflets using the transgastric short-axis view and the fluoroscopic LAO position. Once the position is confirmed, the grasping is performed (in RAO projection and either 4-chamber view or RV inflow-outflow X-plane). The leaflet insertion and the degree of TR reduction are confirmed by multiple TEE views. Once verified, the clip is deployed and TR grade evaluated again before system removal. If the TEE images are of poor quality due to shadowing or challenging anatomy, the use of intracardiac echocardiography can be considered. Leaflet imaging and confirmation of grasping can be achieved with the intracardiac probe in the RA (22). In addition, intracardiac echocardiography guidance allows to perform the procedure under local anesthesia, and this would contribute to the evolution toward a less invasive procedure. Also, the use of fusion imaging (fluoroscopic and echocardiographic views) has been used in challenging cases (23).

Both TOT and BT have also been described in the presence of a transtricuspid CIEDs (24). In a series of 41 consecutive patients treated with the MitraClip system, a transtricuspid CIED was present in 13 of them (32%). In contrast to the large series of patients treated with the edge-to-edge technique (BT used in about 70% of patients) (8, 12), the TOT was used in 77% of patients with CIEDs. Moreover, the CIED extraction (with subsequent implantation of a leadless pacemaker) and thereafter edge-to-edge repair has also been reported (25).

#### Outcomes

The main procedural characteristics, early and mid-term outcomes in the 2 publications including the largest number of patients treated with the MitraClip/TriClip systems to date are summarized in Table 1 (8, 12).

**Table 1:** Clinical, procedural, and follow-up characteristics from the main studies using coaptation devices.

		**MitraClip/TriClip**		**PASCAL**	**FORMA**	
		**Mehr et al. (18) (*n* = 249)**	**Nickenig et al. (8) (*n* = 85)**	**Fam et al. (6) (*n* = 28)**	**Perlman et al. (27) (*n* = 18)**	**Kodali et al. (28) (*n* = 29)**
**Baseline characteristics**						
Age, years		77 ± 9	78 ± 8	78 ± 6	76 ± 10	76 ± 8
Female, *n* (%)		128 (51)	56 (66)	15 (54)	13 (72)	19 (66)
Atrial fibrillation, *n* (%)		183 (74)	78 (92)	26 (93)	16 (89)	24 (83)
NYHA III-IV, *n* (%)		238 (96)	64 (75)	28 (100)	17 (94)	25 (86)
Transtricusid CIED, *n* (%)		74 (30)	12 (14)	1 (3)	3 (17)	n/a
EuroSCORE II, %		6.4 (4-14)	8.6 ± 11	6.2 ± 5.2	9 ± 5.7	8.4 ± 5.3
**Echocardiography**						
LVEF, %		49 ± 14	59 ± 8	59 ± 6	59 ± 9	57 ± 12
TAPSE, mm		16 ± 4	14 ± 0.3	16 ± 3	15 ± 5	14 ± 0.4
TR grade	TR 5+ (%)	n/a	31/84 (37)	12 (43)	11 (61)	n/a
	TR 4+ (%)	129 (52)	24/84 (29)	9 (32)	6 (33)	n/a
	TR 3+ (%)	112 (45)	24/84 (29)	7 (25)	1 (6)	n/a
Maximum gap width, mm		5.3 ± 3.3	n/a	6.9 ± 3	n/a	n/a
Vena contracta width, mm		9.9 ± 4.1	17 ± 0.6	11.4 ± 5	12,1 ± 3.3	16 ± 0.5
EROA, cm^2^		0.70 ± 0.5	0.65 ± 0.3	1.3 ± 2.4	1 ± 0.6	2,2 ± 1.5
Mitral regurgitation ≥ moderate, *n* (%)		108 (43)	0 (0)	n/a	4 (22)	n/a
**Procedural and 30-day outcomes**						
Procedural success, *n* (%)		192 (77)	76/84 (91)	24 (86)	16 (89)	27 (93)
Number of implants, *n*		2 ± 1	2,2 ± 0.8	1,4 ± 0.6	n/a	n/a
Post-procedural tricuspid gradient, mmHg		2.4 ± 1.5	2.12 ± 1.2^*^	1.6 ± 1	n/a	n/a
Procedural Procedural-related Death, *n* (%)		0 (0)	0 (0)	0 (0)	0 (0)	2 (7)
Need for cardiac Surgery, *n* (%)		1 (0.4)	0 (0)	0 (0)	1 (6)	3 (10)
30-day TR grade > moderate, *n* (%)		n/a	36/83 (43)	4/26 (15)	7/16 (44)	n/a
30-day mortality, *n* (%)		n/a	0 (0)	2 (7)	0 (0)	2 (7)
**Follow-up**						
Follow-up time		290 days	6 months	30 days	12 months	30 days
Mortality, *n* (%)		44 (19)	4/84 (5)	2 (7)	0 (0)	N/A
TR grade > moderate, *n* (%)		46 (28)	30/70 (43)	4/26 (15)	12/13 /92)	2 (7)
NYHA I-II, *n* (%)		121 (69)	63/73 (86)	23/26 (88)	11/14 (79)	18/25 (72)

*Values are mean SD or n/N (%)*.

* *At 30 days. CIED, Cardiac implantable electronic device; EROA, effective regurgitant orifice area; EuroSCORE, European System for Cardiac Operative Risk Evaluation; LVEF, left ventricular ejection fraction; NYHA, New York Heart Association; TAPSE, Tricuspid annular plane systolic excursion; TR, Tricuspid regurgitation*.

Mehr et al. reported the results of 249 patients that underwent edge-to-edge repair with the MitraClip system in the TriValve registry (12). The mean age of the patients was 77 ± 9 years, with 51% of women, and a median European System for Cardiac Operative Risk Evaluation score (EuroSCORE) of 6.4% (IQR: 3.9 to 13.9%). Most (94%) patients were in a New York Heart Association (NYHA) class III/IV, and 74% had previous hospitalization for heart failure. Atrial fibrillation and a transtricuspid CIED were present in 74 and 30% of patients, respectively. Concomitant mitral valve clip therapy during the same procedure was performed in half of the patients. Technical success (placement of at least 1 tricuspid clip) and procedural success (defined as TR reduction to grade 2/4 or less) were achieved in 96 and 77% of patients, respectively. On average, 2 ± 1 tricuspid clips were placed, mostly in the anteroseptal position. Patients with TR grade ≥3/4 decreased from 97% at baseline to 23% after the procedure (*p* < 0.001). No procedural deaths were reported and there was no significant tricuspid valve stenosis after clip implantation. On the other hand, one patient with procedural failure was converted to open-heart surgery and there were seven in-hospital deaths. After a median follow-up of 290 (IQR 141-392) days, 72% of patients had an improvement of at least 1 NYHA class grade. Also, TR reduction as evaluated by echocardiography was maintained in 84% of patients. On the other hand, 44 patients (19%) died during the study period, with procedural failure (Hazard ratio: 2.12), absence of sinus rhythm (Hazard ratio: 4.40), and reduced kidney function (Hazard ratio: 1.25 for each reduction of 10 ml/min in the estimated glomerular filtration rate) identified as the factors of increased risk.

The 6-month outcomes of the TRILUMINATE study have been recently published (8). This prospective, multicenter, single-arm, trial aimed to evaluate the safety and performance of the TriClip system for treating severe TR. Patients with systolic pulmonary artery pressure >60 mmHg (determined by echocardiography) or significant mitral regurgitation were excluded. A total of 85 patients with moderate or greater TR undergoing isolated edge-to-edge therapy were included. The average age of the population was 78 years, 33% had prior mitral valve intervention, the mean EuroSCORE was 8.6 and 75% of patients were in NYHA class III/IV. TR severity (evaluated by an independent echocardiography core laboratory) was graded using a five-class categorization: mild, moderate, severe, massive, and torrential (26). Implant success (successful delivery and deployment of at least one clip with the achievement of leaflet approximation and retrieval of the delivery catheter) was achieved in all patients. Overall, 2.2 ± 0.8 clips were used, and most of them were implanted in an anteroseptal position (77%). At least one grade reduction in TR severity was obtained in 91% of patients. At 30 days, single leaflet device attachment (SLDA) was observed in five patients (7%), with no clinical consequences. The echocardiogram performed at 6 months showed that 18/21 (86%) patients with severe TR experienced a reduction to moderate or less TR. Among patients with greater grades of TR at baseline, 12/24 (50%) and 13/43 (43%) with massive and torrential TR reached at least a moderate (or less) grade, respectively. On the other hand, the assessment of mean transtricuspid gradient at follow-up showed an increase from 1.17 ± 0.64 mmHg at baseline to 2.35 ± 1.35 mmHg at 6 months (*p* < 0.001). A total of 6 patients (all of them treated with more than one clip) exhibited a mean gradient >5 mmHg (no specific intervention was required in any of them).

At 6-month follow-up, all patients but 5 survived (95%), and the clinical assessment showed a significant improvement in functional status, with most patients (86%) in NYHA class I-II (*p* < 0.001 vs. baseline). Also, there was a significant increase in exercise capacity, and the mean 6-min walking test (6MWT) distance increased from 278 to 340 meters (*p* < 0.001). In light of the results of the TRILUMINATE study, the TriClip system received the CE mark approbation for minimally invasive tricuspid valve repair.

Whereas the two aforementioned studies showed the feasibility and preliminary efficacy of the MitraClip/TriaClip systems, data regarding long-term improvement in heart failure symptoms and rehospitalization remain scarce. Orban *et al* recently determined the effect of transcatheter edge-to-edge repair (93% MitraClip, 7% Pascal) on the rate of heart failure hospitalization compared with the 1-year period before the intervention (29). A total of 119 patients were included in this retrospective, multicenter study. The main results showed that the mean annual rate of heart failure hospitalization was reduced by 22%, improving from 1.21 to 0.95 hospitalizations per patient/year (*p* = 0.02).

Data regarding the outcomes of the extended clip arms from the MitraClip XTR system for TR reduction are limited. Braun et al. recently reported the acute and short-term outcomes of 31 patients treated with transcatheter edge-to-edge repair using the XTR system at 2 centers (30). Of note, the coaptation gap was ≥7 mm in 52% of patients (theoretically not eligible for NTR system). Overall, the authors demonstrated that TTVI with this device can be successfully performed (procedural success in 87% of patients). However, a high rate of SLDA was observed in patients with a coaptation gap ≥7 (25% of patients), reinforcing the shortcomings regarding edge-to-edge repair in this subset of patients.

Finally, in the only study using the MitraClip and comparing specifically patients with and without the presence of a transtricuspid CIED (a small series of 42 MitraClip recipients, 13 of them with CIEDs), no significant differences in procedural and early outcomes were found between groups (24).

### PASCAL System

The development of the Edwards Pascal transcatheter mitral valve repair (TMVr) system brought a new alternative to edge-to-edge TR treatment (31). The device combines the design features from the MitraClip system (leaflet attachment) and the FORMA device (occupation of the regurgitant orifice with a spacer) (Figure 1). The central spacer increases the maximum span width, which along with the wider length of the device may improve the distribution of the forces applied to the tricuspid leaflets. The latter might reduce the risk of leaflet damage and SLDA. Also, its unique features may potentially be useful to treat patients with larger coaptation gaps (compared to the MitraClip/TriClip system).

#### Device Design and Procedural Planning

The PASCAL TMVr consists of a 10 mm central nitinol woven spacer that acts as a filler in the regurgitant orifice of the atrioventricular valve and is attached to the valve leaflets by two paddles and clasps. The spring-loaded paddles (25 mm width in grasping position) and clasps (10 mm length) are wider than the MitraClip system. The clasps can be operated simultaneously or independently, which can facilitate the capture of the leaflets. The system consists of a 22 French steerable guide sheath, a steerable catheter, and an implant catheter, which contains the implant at the distal end. The handle of the implant catheter controls the deployment of the device and allows the independent movement of the clasps. The device is repositionable and recapturable.

Pre-procedural planning is similar to that used for MitraClip evaluation. However, the bigger size of the device may be a disadvantage in patients with less dilated tricuspid annulus and smaller coaptation gaps. Although the device can be elongated to achieve a narrow profile that could reduce the risk of entanglement, a careful evaluation regarding the subvalvular apparatus (papillary muscles, trabeculations, or prominent moderator band) might be needed to ensure successful device navigation (especially in non-dilated ventricles).

#### Procedural Steps

The TTVI with the edge-to-edge technique using the PASCAL system is currently in its early stages. The framework does not vary compared to the MitraClip/TriClip device, with the procedure performed under general anesthesia with fluoroscopic and TEE guidance.

First, large-bore conventional percutaneous venous access is obtained in the right femoral vein to introduce the 22 French guide sheath. To date, all reported cases using the Pascal system in the context of TTVI have been performed through the femoral approach (6, 31). Once the guide sheath is advanced in the RA and proper orientation is achieved, the implant is advanced into the right ventricle in an open configuration. Afterwards, the implant is carefully retracted and the 2 valve leaflets are grasped either independently or simultaneously. If the leaflets are grasped independently (this might be useful in large coaptation gaps with important tethering), it is important to ensure perpendicular grasp in the second leaflet to avoid valve distortion. Then, the residual TR grade along with the transvalvular gradient is evaluated before device deployment. Finally, the system is retracted and conventional vascular closure is performed.

#### Outcomes

The main baseline characteristics, procedural results, and early outcomes from the recently published first-in-human experience are summarized in Table 1 (6). A total of 28 patients were included. The mean age of the population was 78 ± 6 years and the mean EuroSCORE was 6.5 ± 5.2%. All patients were in NYHA functional class III/IV. The etiology of TR was functional in 23/28 (82%) of patients, with a mean coaptation gap of 6.9 ± 3 mm (patients with coaptation gaps >15 mm, severe leaflet tethering or pacemaker-lead induced TR were excluded). TR grade was 3/5, 4/5, and 5/5 in 7 (25%), 9 (32%), and 12 (43%) patients, respectively as evaluated by the new TR grading classification (26). In 2/28 patients (7%), device implantation was not feasible due to poor quality TEE images (1 patient) and a very large coaptation gap (1 patient). A total of 40 PASCAL devices were implanted (1.4 ± 0.6 per patient), 28 (70%) between the anterior and septal valve tricuspid leaflets and 12 (30%) between the posterior and septal tricuspid leaflets. Independent grasping was used in 90% of implanted devices. Procedural success (defined as implantation of at least 1 device with post-procedural TR grade ≤2, without mortality or conversion to surgery) was achieved in 24/28 (86%) of patients. The intraprocedural echocardiogram showed a slight increase (not clinically significant) in transtricuspid gradients (1.1 ± 0.4 mm Hg vs. 1.6 ± 1.0 mm Hg; *p* < 0.001). Two patients (8%) experienced SLDA during the initial hospitalization and were managed conservatively. At 30-days, most (88%) patients were in NYHA functional class I/II (*p* < 0.001 vs. baseline). Furthermore, the presence of a TR ≥3 was reduced from 100% (28/28) at baseline to 15% (4/26 patients) at 30-day follow-up (*p* < 0.001).

While waiting for future randomized studies, Braun *et al* recently reported the first comparison between the two currently available edge-to-edge systems (32). A total of 120 patients (88 Mitraclip XTR vs. 32 PASCAL recipients) treated in a single center were included. Although the patients in the PASCAL group exhibited higher baseline TR grades, no differences in procedural success were found between groups (86.4% in the MitraClip XTR group vs. 90.6% in the PASCAL group, p=0.75). SLDA was observed in 10/88 (11%) patients in the MitraClip XTR group and in 2/32 (6%) patients in the PASCAL group (*p* = 0.5). Thus, this first non-randomized data showed comparable results with both coaptation devices.

### FORMA

The FORMA repair system aimed to improve the tricuspid valve coaptation by occupying the regurgitant orifice with a spacer, which provided a surface for native leaflets coaptation to reduce the orifice area. The device consisted of a spacer that was advanced through a rail anchored at the septal portion of the right ventricle (RV) apex (Figure 1). Of note, the FORMA system is not currently available for medical use.

#### Device Design and Procedural Planning

The FORMA spacer device is a foam-filled polymer balloon that passively expands via holes in the spacer shaft. A steerable delivery catheter was used to deliver the rail system to the ideal location in the RV apex. The presence of a CIED did not contraindicate the procedure.

#### Procedural Steps

**Figure 4:**
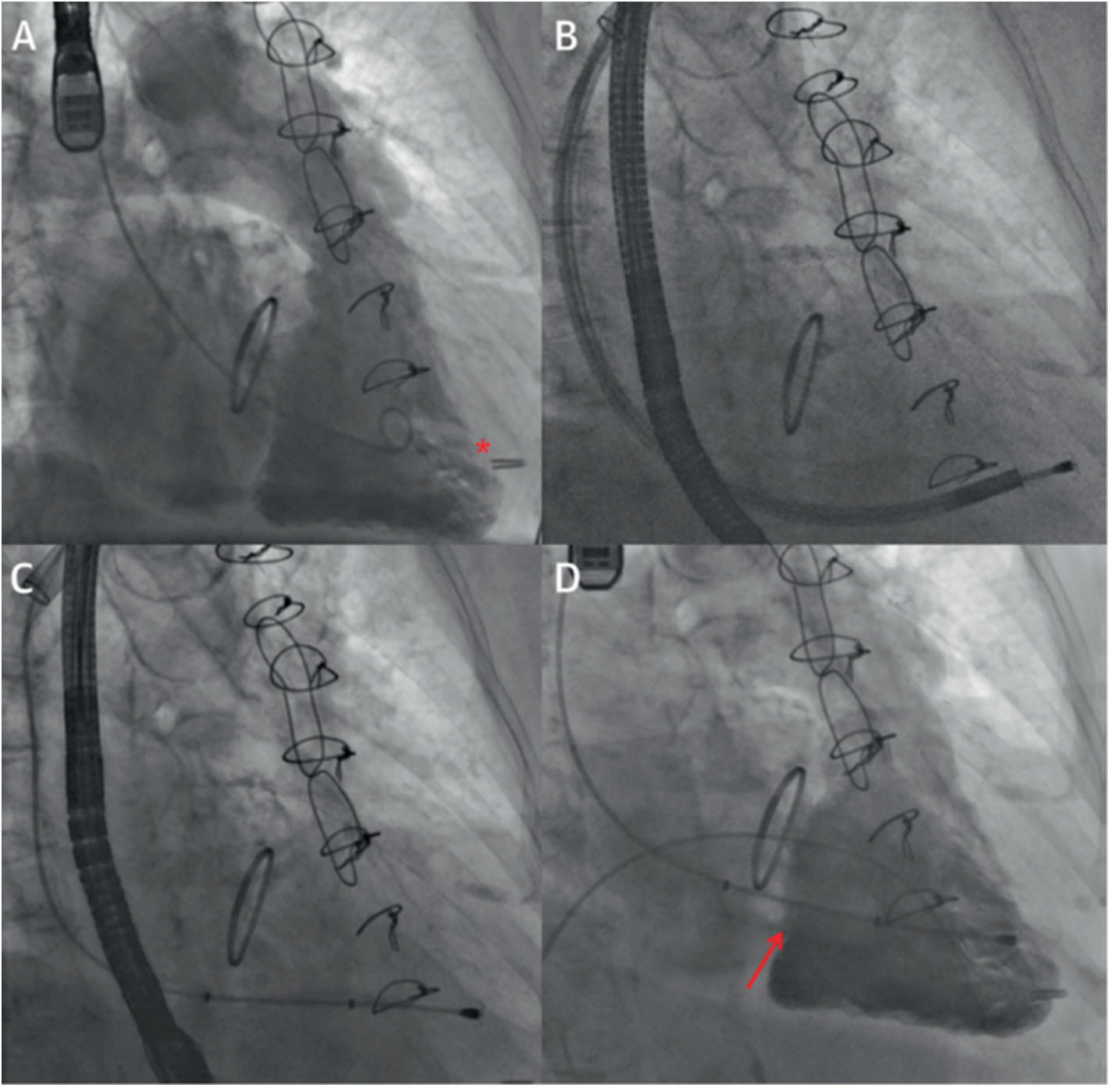
Intraprocedural fluoroscopic images in transcatheter tricuspid valve intervention using the FORMA system. **(A)** Right ventriculography to locate the tricuspid annular plane and identify the ideal anchor location (red asterisk) on fluoroscopy. **(B)** Right ventricular anchoring via a steerable delivery catheter. **(C)** Device positioning in the tricuspid annular plane. **(D)** Final right ventriculography showing the device in correct position (red arrow) and reduction of tricuspid regurgitation from baseline. Reproduced with permission from Campelo-Parada et al. (33).

Following left axillary or subclavian vein access, a large-bore sheath (20 French for the 12-mm device, 24 French for the 15 and 18-mm devices) was advanced to the left innominate vein/superior vena cava junction. After right ventriculography, the rail was anchored to the right ventricle and the spacer was advanced over the rail and placed within the tricuspid valve. Finally, the device was locked proximally, with the excess rail length placed in a subcutaneous pocket. The main steps of the procedure are depicted in Figure 4 (33).

#### Outcomes

Table 1 summarizes the main available data regarding the FORMA device (27, 28). Two main patient cohorts have been reported after the first-in-human cases (33). These two studies represent a total of 47 patients; 18 treated under an international (Canada and Europe) compassionate clinical use program (27) and 29 in the US early feasibility (EFS) trial (28).

In the multicenter international study (27), 2 procedural failures were reported (RV perforation and device dislocation, respectively). All patients were alive at 30-days, and a total of 14 patients had completed the 1- year follow-up. The NYHA functional class improved (by at least 1 grade) in 86% of them, and the median increase in 6MWT distance was 84 meters between baseline and 1-year follow-up (*p* = 0.032).

In the EFS trial (28), 2 procedural failures due to RV perforations were reported. In addition, there were two device explantations (detachment of the anchor and endocarditis, respectively), and 2 procedural procedural-related deaths occurred, due to RV perforation and as a consequence of the surgical explant, respectively. On the other hand, the results at 30 days showed improvements in NYHA functional class (28% of patients in NYHA class ≥III vs. 84% at baseline, *p* = 0.0002) and in the Kansas City Cardiomyopathy Questionnaire (KCCQ, increase by 29 points, *p* < 0001).

The echocardiographic evaluation at 30 days showed a relevant reduction in TR severity in the 2 aforementioned studies. In the EFS trial, the Core Lab assessment showed significant reductions in TR grade in the 25 available patients, with a quantitative effective regurgitation orifice area reduced by 49% (from 2.1 ± 1.8 cm^2^ at baseline to 1.1 ± 0.9 cm^2^ at 30-days, *p* = 0.012). In the compassionate clinical use cohort, the echocardiographic evaluation showed a reduction in the grade of TR in all but one patient. However, the effective regurgitation orifice area and vena contracta assessments did not improve between 30 days and 1 year (27). Despite the latter, the reduction in TR severity seemed to be sufficient for determining a significant clinical improvement.

Finally, Asmarats et al. reported the long-term outcomes from the multicenter international cohort (34). At a median follow-up of 3 years, 4/17 (24%) patients had died (3 from terminal heart failure, 1 from sepsis), and 3/17 (18%) patients required re-hospitalization for heart failure. There were 1 device-related thrombosis and 1 pulmonary embolism (both of them with sub-therapeutic oral anticoagulation targets). At 2-year follow-up, 66% of patients were in NYHA functional class I/II (vs. 7% at baseline, *p* < 0.001), and both 6MWT and KCCQ improved significantly. However, two-thirds of the patients remained with a significant TR, with no significant changes in the effective regurgitation orifice area (baseline: 0.92 cm^2^, follow-up: 0.77 cm^2^; *p* = 0.52). These findings suggested that despite the improvements in 30-day echocardiographic parameters, a deleterious RV remodeling could have occurred at long-term follow-up. The patients included in this cohort had long-lasting TR with severe leaflet tethering, likely contributing to TR recurrence as demonstrated in surgical cohorts (35, 36).

## Conclusions

In conclusion, TTVI using coaptation devices is a promising technique that has demonstrated procedural feasibility, early safety, and satisfactory mid-term outcomes. However, some issues as patient and device selection, optimization of intraprocedural results, and long-term data should be addressed in future studies. If the preliminary efficacy data are confirmed in further studies, this therapy will represent a paradigm shift in the management of chronic TR.

## Author Contributions

All authors listed have made a substantial, direct and intellectual contribution to the work, and approved it for publication.

## Conflict of Interest

JR-C has received institutional research grants from Medtronic and Edwards Lifesciences. The remaining authors declare that the research was conducted in the absence of any commercial or financial relationships that could be construed as a potential conflict of interest. The reviewer FP declared a past co-authorship with one of the authors GM-C to the handling editor.

## Abbreviations

6MWT: 6-minute walking test
BT: Bicuspidization technique
CIED: Cardiac implantable electronic device
CDS: Clip delivery system
EFS trial: early feasibility trial
EuroSCORE: European System for Cardiac Operative Risk Evaluation
LAO: Left anterior oblique
IQR: Interquartile range
KCCQ: Kansas City Cardiomyopathy Questionnaire
NYHA: New York Heart Association
RA: right atrium
RAO: Right anterior oblique
RV: Right ventricular
SLDA: Single leaflet device attachment
SGC: Steerable guide catheter
TMVr: Transcatheter mitral valve repair
TOT: Triple-orifice technique
TR: Tricuspid regurgitation
TTVI: Transcatheter tricuspid valve intervention
TV: Tricuspid valve.
